# What do we know about long-term effects of bariatric surgery?

**DOI:** 10.1093/bjs/znac327

**Published:** 2022-09-27

**Authors:** Dag Holmberg, Jesper Lagergren

**Affiliations:** Upper Gastrointestinal Surgery, Department of Molecular medicine and Surgery, Karolinska Institutet and Karolinska University Hospital, Stockholm, Sweden; Upper Gastrointestinal Surgery, Department of Molecular medicine and Surgery, Karolinska Institutet and Karolinska University Hospital, Stockholm, Sweden; School of Cancer and Pharmaceutical Sciences, King’s College London, and Guy’s and St Thomas’ NHS Foundation Trust, London, UK

Bariatric surgery is an effective treatment for morbid obesity, resulting in rapid, pronounced, and sustained weight loss, improved quality of life, and prolonged life expectancy^1^. Obesity-related disorders, such as diabetes, hypertension, and hyperlipidaemia, typically improve or even resolve after bariatric surgery. The increasing prevalence of obesity combined with the proven benefits of surgery have led to widespread implementation, and bariatric surgery is now among the most common gastrointestinal operations in the Western world. Laparoscopic gastric bypass and sleeve gastrectomy are presently the two most frequently performed bariatric procedures.

It should also be acknowledged that a considerable proportion of operated patients experience side-effects, which often manifest many years after the procedure, including various gastrointestinal disorders and need for endoscopic or surgical reinterventions, for example because of internal hernia and marginal ulcers^2^.

Malabsorption of nutrients or endocrine dysregulation can cause medical conditions after bariatric surgery. In gastric bypass, most of the stomach and the first portion of the small bowel are disconnected from the passage of food, frequently resulting in malabsorption of essential substances absorbed in these parts of the gastrointestinal tract, including iron, calcium, folate, vitamin A, vitamin B1, vitamin B12, and vitamin D. Although many patients are routinely put on supplements in order to avoid deficiencies after surgery, nutritional disorders are five times more common in patients who have undergone bariatric surgery than in unoperated patients^2^. Malnutrition seems to be more pronounced after gastric bypass, but could also be an issue after sleeve gastrectomy^2^. Long-term data from a recent observational study^3^ indicate that the risk of manifest anaemia owing to iron or vitamin B12 deficiency is increased almost three-fold after bariatric surgery. Rapid weight loss and malabsorption of calcium and vitamin D can lead to reductions in bone mineral density, which might result in osteoporosis and increased risk of bone fractures^4^. Vitamin A deficiency is less common, but might in rare cases cause ocular symptoms.

Bariatric surgery leads to an unphysiologically rapid transit of recently ingested food to the small bowel, and undigested carbohydrates might cause late dumping, characterized by symptoms of hypoglycaemia 1–3 h after a meal. Late dumping occurs in more than one-fifth of patients after gastric bypass. The condition rarely develops earlier than 6 months after surgery. Late dumping can often be managed with dietary changes, such as a diet low in carbohydrates and rich in protein and fibre, reduced portion sizes, and lying down after a meal to slow down the initial food passage^5^. Patients with refractory late dumping might require longstanding medication, or, as a last resort, reversal of the gastric bypass or resection of the pancreatic tail.

Patients who have undergone gastric bypass are at increased risk of developing postoperative abuse of alcohol^6^. The mechanisms are unclear, but the increased speed of transit and absorption of alcohol to the bloodstream increases the potency of ingested alcohol, which might contribute to overconsumption. Other potential reasons for substance abuse after bariatric surgery are psychiatric co-morbidity, such as depression and anxiety, as well recurring or remaining somatic health issues related to the bariatric procedure. Thus, although overall mortality is decreased after bariatric surgery, mortality from suicide is increased^7^.

Obesity increases the risk of several types of cancer. Studies have indicated that weight loss through bariatric surgery decreases the risk of several obesity-related cancer types, particularly breast and endometrial cancer. There is more controversy regarding colorectal cancer, because some large-scale observational and experimental studies have indicated an increased risk of developing such tumours after bariatric surgery. However, the results are conflicting, and the relationship between bariatric surgery and colorectal cancer risk is uncertain^8^.

Gastro-oesophageal reflux disease (GORD), which is common in patients with obesity, often improves with weight loss. Consequently, many patients with GORD report fewer reflux symptoms after bariatric surgery and may quit antireflux medication. The benefit seems to be most apparent after gastric bypass; in one study^9^, 62.8 per cent of patients reported resolved GORD within 6 months of surgery, whereas 15.9 per cent reported resolution of GORD and 8.6 per cent developed *de novo* GORD after sleeve gastrectomy. A recent meta-analysis^10^ found that, after sleeve gastrectomy, 11.6 per cent of patients develop Barrett’s oesophagus, a premalignant condition of oesophageal adenocarcinoma that requires endoscopic surveillance. Because most patients undergoing bariatric surgery are young and female, which are protective factors for oesophageal adenocarcinoma, the absolute risk of oesophageal adenocarcinoma is still low in these patients. Among older and male patients, the large proportion with Barrett’s oesophagus after sleeve gastrectomy may pose a larger issue. Barrett’s oesophagus is often unrelated to GORD symptoms, and it is possible that older and male patients who undergo sleeve gastrectomy may benefit from later endoscopic screening to detect the condition.

Despite major health benefits, bariatric surgery is associated with risks of several late-occurring and serious medical conditions that may be difficult to treat, often requiring lifestyle changes, medication, and additional surgery. Understanding these risks is important for healthcare providers and patients.
